# LC-Orbitrap-MS/MS Analysis of Chosen Glycation Products in Infant Formulas

**DOI:** 10.3390/molecules30132753

**Published:** 2025-06-26

**Authors:** Aleksandra Damasiewicz-Bodzek, Magdalena Szumska, Agnieszka Nowak, Sławomir Waligóra, Beata Pastuszka, Kamila Stopińska, Beata Janoszka

**Affiliations:** 1 Department of Chemistry, Faculty of Medical Sciences in Zabrze, Medical University of Silesia, 40-055 Katowice, Poland; aleksandra.bodzek@sum.edu.pl (A.D.-B.); mszumska@sum.edu.pl (M.S.); swaligora@sum.edu.pl (S.W.); bjanoszka@sum.edu.pl (B.J.); 2 Research and Implementation Center Silesia LabMed, Medical University of Silesia, 40-752 Katowice, Poland; beata.gierat@sum.edu.pl; 3 Clinical Department of Dermatology, Faculty of Medical Sciences in Zabrze, Medical University of Silesia, 40-055 Katowice, Poland

**Keywords:** infant formula, glycation, AGEs, CML, CEL, furosine, GOLD, MOLD

## Abstract

When breastfeeding is not possible, infant formulas may be used instead of human milk. However, harmful advanced glycation end-products (AGEs) may be formed during thermal processing of infant formulas. The exposure to AGEs at such an early age can lead to chronic diseases in the future. Therefore, the aim of this study was to develop a sensitive method to determine the content of AGEs in infant formulas. Twenty commercial infant formulas (initial and follow-on) in liquid and powder form were investigated using liquid chromatography with tandem mass spectrometry (LC-MS/MS) with a multistep sample pretreatment procedure. Five selected glycation products were analyzed: N^ε^-carboxyethyllysine (CEL), N^ε^-carboxymethyllysine (CML), furosine, glyoxal lysine dimer (GOLD), and methylglyoxal lysine dimer (MOLD). The mean contents of the tested glycation products did not differ significantly between the initial and follow-on formulas. No significant differences were found in the concentrations of the analyzed compounds from different manufacturers. However, the liquid formulas contained significantly more CML. The estimated dietary exposure to the tested compounds was in the range of 42.5–92.6 μg/day, except for furosine (almost 2 mg/day). The developed method enabled the determination of selected AGEs in complex matrices such as infant formulas. Consumption of liquid infant formulas can result in higher exposure to some AGEs.

## 1. Introduction

Glycation is usually described as a non-enzymatic reaction between amino acids and carbonyl compounds (including saccharides). Other terms used to describe this reaction are glycoxidation (to emphasize the relationship between it and the oxidizing environment) and Maillard reaction (more commonly used in the field of food safety research).

While the initial definition—a reaction between amino acids and carbonyl compounds—seems to be simple, the reaction itself is very complex. Attempts to divide it into early, intermediate, and advanced stages have been made, and this division is reflected in the naming of compounds taking part in it: early glycation products (EGPs), intermediate glycation products (IGPs), and advanced glycation end-products (AGEs) [1,2]. Among EGPs, there are unstable imines (Schiff bases) that quickly undergo Amadori rearrangement. Furosine is considered a product of the early stages of the reaction. IGPs include furfurals and a plethora of dicarbonyl compounds, such as glucosone, 1-deoxyglucosone (1-DG), and 3-deoxyglucosone (3-DG). Reactions of cleavage, cyclisation, and isomerization occur, leading to the formation of AGEs. Examples of such compounds are N^ε^-carboxyethyllysine (CEL), N^ε^-carboxymethyllysine (CML), glyoxal lysine dimer (GOLD), and methylglyoxal lysine dimer (MOLD) [1,3,4,5].

However, the division between substrates and products of glycation is not clear. For example, methylglyoxal (MGO) can act as the carbonyl substrate at the beginning of the reaction, as an intermediate that reacts with arginine or lysine, or even become a product, as it can arise from 3-DG degradation [6].

The course of glycation in food products is different from its course in the human body. Unprocessed food, especially of animal origin, already contains some AGEs. Nevertheless, foodstuffs often undergo thermal treatment, which results in the formation of new AGEs. They also differ by water content and pH. These factors affect the outcome of the reaction [4,7]. The most advanced products of glycation in food (advanced Maillard reaction products—advanced MRPs) are brown polymeric pigments called melanoidins. They are responsible for the desirable flavor and dark coloration of products such as baked bread and roasted coffee [5].

Breastfeeding is a gold standard of infant feeding, especially during the first 6 months of life. It is recommended by the Polish and the European Society for Paediatric Gastroenterology, Hepatology and Nutrition, and the World Health Organization [8,9,10]. However, breastfeeding is not always possible. In such cases, infant formulas are used. These formulas are divided into initial formulas (Stage 1, used between the 0th and 6th month of life) and follow-on formulas (Stage 2, used between the 6th and 12th month of life). Infant formulas are a type of food designed to imitate human milk. Nonetheless, the compositions of human milk and infant formulas differ; what is more, some formulas have a decreased content of lactose or allergenic proteins in order to better respond to infants’ needs. Formulas are made of proteins or protein hydrolysates derived from cow’s milk, goat’s milk, or soy. They have strictly controlled contents (caloricity, maximum and minimum amount of nutrients and additives) [11]. Infant formulas undergo thermal treatment during production—high temperature may enhance the synthesis of AGEs [12,13]. For example, furosine is considered an indicator of heat-induced damage to a product [4]. Interestingly, there are no regulations regarding the AGE content in infant formulas. The European Food Safety Authority (EFSA) requires manufacturers to report only levels of CML and furosine in their products, but there is no defined upper limit [14,15,16].

Dietary AGEs (dAGEs) are known to pass through the intestinal wall. It is estimated that 10% of ingested AGEs enter circulation, and one-third is eliminated through urine within 48 h. The remaining two-thirds may be either removed over a longer period of time or incorporated into native body proteins [17]. It is known that the absorption and metabolism of dAGEs is a complex process depending on many factors. Despite the obvious challenges in the estimation of the ratio of incorporated to ingested AGEs, such attempts are made [18]. Exposure to dAGEs in early life stages is of particular interest [19]. There is concern that immature gastrointestinal tract structures, newly developing intestinal microbiota, and immature detoxication systems may contribute to greater exposure to dAGEs in infants, and therefore lead to greater consequences in later life than in adults.

As explained above, humans are exposed to dietary AGEs, but glycation products can also be formed within the body under physiological and pathological conditions. Glycation products are not neutral to the human body.

Glycation products may be recognized by the immune system as neo-epitopes [20]. Some glycation products (such as pentosidine, threosidine, MOLD, and GOLD) are able to crosslink protein molecules. These crosslinks negatively affect tissue elasticity, which is especially unfavorable in the case of proteins characterized by a long half-life [3,21]. Glycated proteins are more resistant to intracellular proteasomal degradation [22] and enzymatic digestion in the gastrointestinal tract, decreasing the bioavailability of amino acids [23]. Ascorbic acid may be depleted during this reaction, reducing the nutritional value of the product [3]. Glyceraldehyde-derived AGEs are known to be cytotoxic [24]. AGEs activate a pro-inflammatory receptor for advanced glycation end-products (RAGE) and induce reactive oxygen species formation [6,25]. Thus, some authors use the term toxic AGEs (TAGEs) or glycotoxins to describe the highly reactive glycation products [7,24]. According to Song et al., AGEs may contribute to insulin resistance to some degree [26]; however, such a causation was not observed in a study by Klenovics et al. that examined infants over a period of 7.5 months [13]. Mericq et al. noted that a high concentration of maternal serum methylglyoxal derivatives is reflected in higher infant insulin levels. AGE serum concentration correlates negatively with adiponectin level in infants. Additionally, the authors are concerned about a rapid increase in dietary AGE exposure during the gradual replacement of breast milk with infant formula and solid foods [27]. Unfortunately, the outcomes of high early exposure to AGEs in human infants are studied less extensively than in laboratory animals. In animal studies, it has been concluded that even prenatal exposure to AGEs negatively affects postnatal development [28].

Considering the above, the presence of AGEs in infant formulas is an unfavorable phenomenon. Manufacturers should aim to reduce their content in products. Therefore, the objective of the study was to examine the content of glycation products in infant formulas and to estimate dietary exposure to potentially harmful AGEs in infants. A state-of-the-art method [29]—liquid chromatography with tandem mass spectrometry (LC-MS/MS)—was used.

## 2. Results

### 2.1. Development of the Chromatographic Method

The initial aim of the study was to optimize chromatographic separation of a standard mixture containing furosine and lysine derivatives (CML, CEL, GOLD, and MOLD). At this stage, the Poojary et al. [30] protocol for the analysis of advanced glycation end-products in food and biological matrices was used. Modifications in the mobile phase composition and gradient duration were made to the original procedure to achieve a higher resolution of chromatographic separation for selected glycation products. Based on the literature information [30] and our analytical experience, various options of gradient were tested. The best resolution for the selected glycation products was obtained with the mobile phase composition presented in Section 4.5. In the process of method optimization, the separation and detection of methylglyoxal-derived hydroimidazolone (MG-H1), N^ε^-carboxymethylarginine (CMA), and N^ε^-carboxyethylarginine (CEA) was also achieved. Unfortunately, the recovery rates and repeatability for the compounds mentioned above were not sufficiently satisfactory. Thus, we were not able to apply the developed sample pretreatment method to analyze these particular glycation products.

**Figure 1:**
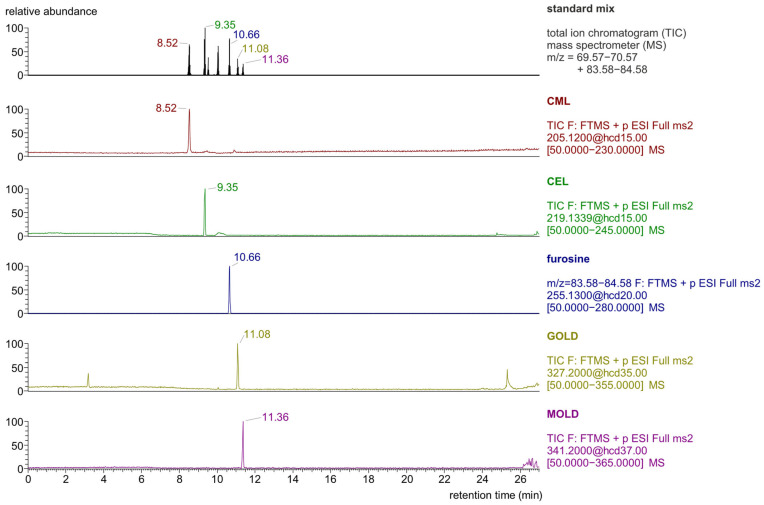
LC-Orbitrap-MS/MS chromatograms recorded in total ion mode (top) and PRM mode for a standard mixture of glycation products (625 ng/mL, injection volume 1 μL).

**Figure 2:**
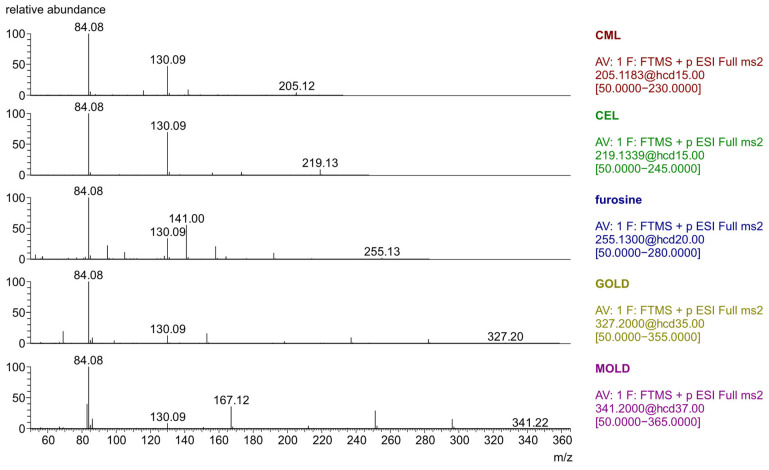
Mass spectra of glycation product standards recorded in PRM mode, with collision energies presented in Table 1.

The determination of selected glycation products using the LC-MS/MS technique was performed in parallel reaction monitoring (PRM) mode. Figure 1 and Figure 2 show examples of total ion and PRM chromatograms recorded during the analysis of the standard mixture.

Using PMR acquisition at various collision energy values, the product ion of the highest intensity was selected. In our study, this product ion was 84.0806. The optimal collision energies used to obtain product ions from precursor molecular ions [M + H]^+^ corresponding to the maximum intensity for the analytes are shown in Table 1. The parameters for the optimal MS detection were also chosen based on studies on AGE analysis in various biological samples, including infant formulas [30,31,32,33].

Quantitative analysis of 5 glycation products was performed on the basis of linear calibration graphs recorded in the range of 62.5 ng/mL to 1250 ng/mL (Figure S1), using 1 µL of standard mixture injected into the column. Calibration curve equations and correlation coefficients *r* for the analyzed compounds are presented in Table 2. For CML, CEL, furosine, and MOLD, the coefficients were around 0.99. The weakest correlation, below 0.9, was recorded for the GOLD calibration curve plotted in the range of 62.5 ng/mL to 1250 ng/mL. Outliers were noted for concentrations of this compound at 800 ng/mL and above. Accordingly, another curve was generated for GOLD in the range of 62.5 ng/mL to 625 ng/mL, for which the correlation coefficient r was 0.982 (Figure S1). This curve was used to calculate the concentration of GOLD in infant formula samples.

The limits of detection (LOD) were determined using the stepwise dilution method of standard solutions, taking them as a signal-to-noise (S/N) ratio of 3. A value of 3 times the limit of detection (LOD) was taken as the limit of quantification (LOQ) for an injection volume of 1 µL. Based on these data, the LOD for all analyzed compounds was 20 ng/mL and the LOQ was 60 ng/mL in standard solutions. This corresponds to 0.5 μg/mL and 1.5 μg/mL in infant formula, from which AGEs were extracted according to the description in Section 4.4. The injection volume of the infant formula extracts was also 1 µL.

The recovery levels were determined using a milk powder formula with the lowest detected amounts of the analyzed compounds. The milk sample was enriched with standards of selected AGEs at three concentration levels (125 ng/mL, 625 ng/mL, 1250 ng/mL). Six analyses were performed at each level. After the enrichment, the full procedure of sample pretreatment (Section 4. Materials and Methods) was carried out. Then, the analytes were determined using LC-Orbitrap-MS/MS in PRM mode.

The recovery calculations were based on the following equation:

$$recovery=\frac{c_{1}-c_{2}}{c}\cdot 100\%$$

where $c_{1}$ is the analyte concentration in the milk sample containing added standards (ng/mL), $c_{2}$ is the determined concentration in the milk sample (ng/mL) not enriched with standards, and c is the amount (ng) of standard added to the sample.

Recovery data and the relative standard deviation (RSD%) are shown in Table 3. They ranged from 31.1% to 121.7%. Recoveries obtained in our study corresponded to literature data except for furosine [30]. A very low recovery (31.1%) was noted for furosine at the lowest spiking level, which may suggest an effect of the sample matrix on the recoveries. For the two remaining spiking levels, which were closer to the levels of furosine in the infant formula extracts (Supplementary Table S2), the recovery was much higher (about 70%).

The repeatability (intraday precision) of the method was calculated as RSD% of the data obtained on the same day (n = 6) for samples enriched with standards. These ranged from 0.93% to 1.42%. The intermediate precision (inter-day precision) of the method was also calculated as RSD% over 5 days and ranged from 3.41% to 6.80%.

### 2.2. Infant Formula Samples

The determined concentrations of the analyzed glycation products in the initial formulas (IFs, Stage 1 formulas, samples A1–J1) and follow-on formulas (FFs, Stage 2 formulas, samples A2–J2) are presented in Table 4.

The mean contents of the AGEs tested did not differ significantly between the initial and follow-on formulas (*p*-values 0.19–0.91). Comparisons between the content of the tested compounds in the initial and follow-on formulas of a given manufacturer also did not show any statistically significant differences (*p*-values 0.11–0.75). The basic parameters of the descriptive statistics for the compounds studied are presented in Table 5.

Supplementary Figures S2–S6 show examples of PRM chromatograms recorded during the analysis of an extract of a ready-to-serve liquid infant formula sample (H2). The selected LC-MS/MS analysis parameters in PRM mode (Table 1) enabled the detection and determination of selected glycation products in infant formula, which is a complex food matrix. However, in the case of our investigation, some compounds were detected at very low concentrations, and sometimes the precursor ion was not visible in the PRM mass spectrum. This occurs in the case of GOLD and MOLD (Figures S4 and S5). Moreover, due to the possible presence of other compounds in the samples and the relative abundance parameter (%) of the scale, the product ion may appear low in intensity (see GOLD, Figure S4).

## 3. Discussion

Our study provides data on the concentrations of selected glycation products present in infant formulas available on the Polish market. Based on these results, it can be noted that there are minor differences in the content of the analyzed glycation products between infant formulas provided by various manufacturers. Furthermore, Stage 1 initial formulas and Stage 2 follow-on formulas are very similar in this regard. None of the samples differed in a statistically significant manner. This is most likely due to the similarities in their nutrient content (Table 6).

Four of the compounds analyzed in our study—CML, CEL, GOLD, MOLD—are derivatives of lysine, an essential amino acid [3]. Additionally, the concentration of furosine was determined, as it is a known marker of thermal treatment in food products [4]. In the following discussion, our results were compared to those obtained using MS-based methods. Other commonly used methods, such as the enzyme-linked immunosorbent assay (ELISA), have been reported to provide much higher results (up to 17-fold higher) [34]. Importantly, the studies cited in the following section often apply different hydrolysis and glycation product isolation procedures.

In our study, the concentration of CML ranged from 1.82 to 5.96 μg/mL of the ready-to-serve milk. The determined mean concentration was 258.25 mg/kg protein in IF and 225.85 mg/kg protein in FF. These values are within the range reported by other authors (17–405 mg/kg protein) for infant formulas [12,34]. In the study performed by Wei et al., CML concentration in infant formulas was relatively low—0.83–31.73 mg/kg; however, their sample preparation procedure was significantly different from ours [35]. Importantly, the mean CML concentration in the liquid formulas studied by us was significantly higher than in the powdered formulas (4.36 ± 1.43 μg/mL vs. 2.81 ± 0.99 μg/mL FF, *p* < 0.05). Delatour et al. observed a similar trend: 75.6 ± 48.5 ng/mg protein in powder (75.6 ± 48.5 mg/kg protein) and 153 ± 40 ng/mg protein in liquid (153 ± 40 mg/kg protein) [34].

The CEL concentration was 0.81–1.85 μg/mL of ready-to-serve milk in our study. Xie et al. reported CEL concentrations of approximately 5–58 mg/kg protein [12]. Our mean results (118.1 mg/kg protein for IF and 121.4 mg/kg protein for FF) exceeded this range. Wei et al. reported a CEL concentration of 0.72–7.51 mg/kg [35].

The concentration of furosine ranged from 39.27 to 102.35 μg/mL in our study. Its amount was almost 9 times higher than the sum of other AGEs. The mean furosine concentration was 5069.7 mg/kg of protein in IF and 5519.9 mg/kg of protein in FF. We noticed a tendency for higher concentrations in FF vs. IF (mean 77.72 μg/mL vs. 69.10 μg/mL, respectively), but this difference was not statistically significant. This may be caused by some differences in the composition of the formulas, mainly in the case of proteins and carbohydrates. FF may have a higher carbohydrate content than IF, and this was true for our samples (mean carbohydrates 7.42 ± 0.32 g/100 mL in IF and 8.05 ± 0.23 g/100 mL FF, *p* < 0.005).

Our results are higher than the range of 152–2750 mg/kg of protein presented by Xie et al. in their work [12]. In contrast, Martysiak-Żurowska et al. detected furosine at levels of 9319–15,509 mg/kg of protein in formulas available on the Polish market, which is two to three times higher than the levels found in our study. However, it is worth noting that this study was performed in 2007 using a higher concentration of HCl, a higher temperature during isolation, and UV detection instead of mass spectrometry [36].

While there are many reports on CML, CEL, and furosine, research on compounds such as GOLD and MOLD is less common. The assessment of GOLD and MOLD concentrations contributes to the novelty of our research. In our study, GOLD ranged from 1.51 to 1.67 μg/mL milk. The mean concentration in IF was 117.4 mg/kg protein, and in FF it was 111.5 mg/kg protein. Akıllıoğlu et al. detected no GOLD in liquid formulas, and 32 mg/kg protein of this compound in powder formulas [37]; however, we did not observe such a difference in our samples. MOLD in our samples was at a concentration of 1.66–1.84 μg/mL milk. IF contained 130.6 mg/kg protein and FF 126.4 mg/kg protein. Akıllıoğlu et al. detected MOLD in infant formulas, too, but could not quantify it [37].

According to the literature data, the initial stages (pasteurization and homogenization) of liquid and powder formula production are similar. Significant differences in the thermal treatment process occur at the stages of adapting the product to its final physical state. Liquid infant formulas may be subjected to ultra-high-temperature (UHT) treatment to ensure sterility, which allows for an extended shelf life and product stability without the need for refrigeration. Temperatures in the range of 135–150 °C are applied for a few seconds. In contrast, powdered formulas are produced using the spray drying method, during which the milk is heated to temperatures in the range of 180–200 °C for a few seconds. In spray drying, the temperature of the milk particles remains lower (typically 70–80 °C) due to the cooling effect of water evaporation [38,39]. Interestingly, the commonly used heat treatment marker—furosine—did not differ significantly in IF vs. FF and liquid vs. powder comparisons in our study. The only noticeable difference was in CML concentration in liquid vs. powder formulas. Li et al. showed that UHT processing of milk leads to the formation of glycation products, including CML [40]. Importantly, differences related to the heat treatment occurring in the production process of powdered and liquid formulas are not the only factor that determines the amount of AGEs in infant formulas. Other factors include product moisture, pH, chemical composition (e.g., the content of reducing sugars, protein, fat, antioxidants, and pro-oxidants), and storage conditions [38].

The data obtained could be used to estimate the exposure to the analyzed glycation products in infants. Using mean milk intake calculated by Rios-Leyvraz et al. (670 mL per day) [41], the following daily consumption estimates can be made: CML, 2.24 mg/day; CEL, 1.11 mg/day; furosine, 49.18 mg/day; GOLD, 1.06 mg/day; and MOLD, 1.19 mg/day.

## 4. Materials and Methods

### 4.1. Infant Formula Samples Preparation

A total of 20 commercial infant formulas in liquid and powder form, available on the Polish market, were analyzed. The sample set included Stage 1 initial formulas, intended for infants from birth to 6 months (samples A1–J1), and Stage 2 follow-on formulas, intended for infants older than 6 months as part of a weaning diet (samples A2–J2). All tested formulas were within their expiration date and were sourced from different manufacturers. The energy value and content of the basic nutrients are presented in Table 6. Detailed information on the infant formula composition is provided in Supplementary Table S1.

### 4.2. Chemicals and Reagents

All standards, namely CEL (mixture of two diastereoisomers), CML, GOLD (acetic acid salt), MOLD (acetic acid salt), and furosine (hydrochloride salt), were purchased from Iris Biotech GmbH (Marktredwitz, Germany). Hydrochloric acid (37%, analytical grade) was obtained from Chemland (Katowice, Poland). Acetonitrile (LiChrosolv^®^ hypergrade, suitable for LC-MS), water (LiChrosolv^®^ hypergrade, suitable for LC-MS), and perfluoropropionic anhydride (99%) (PFPA) were purchased from Sigma-Aldrich (Darmstadt, Germany). Ultrapure water was produced using a Polwater DL-2 series deionizer supplied by Labopol-Polwater (Kraków, Poland). Pierce™ LTQ Velos ESI Positive Ion Calibration Solution (Thermo Fisher Scientific, Rockford, IL, USA) was used to tune and calibrate the mass spectrometer.

### 4.3. Preparation of Stock Solutions and Calibration Curves for LC-MS/MS Analysis

Stock solutions of the analytical standards (CML, CEL, furosine, GOLD, and MOLD) were prepared by dissolving each compound in LC-MS-grade water (LiChrosolv^®^ hypergrade). Stock solutions of CML and CEL were prepared at a concentration of 50 mg/mL, while furosine, GOLD, and MOLD were prepared at 10 mg/mL. A working solution containing all the standards was prepared at an initial concentration of 25 μg/mL. This working solution was serially diluted to obtain concentrations ranging from 1250 ng/mL to 62.5 ng/mL, which were used to construct the calibration curves.

### 4.4. Isolation of Glycation Products from Infant Formulas

Powdered infant formulas were reconstituted according to the manufacturers’ instructions included on the packaging. To initiate acid hydrolysis, 40 μL of each sample was transferred to 2 mL amber glass vials and mixed with 200 μL of 6.6 M hydrochloric acid dissolved in deionized water. To ensure homogeneity, the samples were vortexed for 10 s at 3000 rpm. The headspace of each vial was purged with nitrogen (≥99.99% purity) at a flow rate of 50 mL/min for 1 min to minimize the oxidative degradation of the analytes. The vials were hermetically sealed and incubated in a temperature-controlled heating furnace at 90 °C (±1 °C) for 24 h. Following hydrolysis, the samples were evaporated to dryness under a gentle stream of nitrogen (20 mL/min) at 40 °C for 25 min. The resulting residues were reconstituted in 1 mL of LC-MS-grade water and vortexed at 3000 rpm for 1 min. The samples were ultrasonicated for 30 min in a Sonic 6 temperature-stabilized bath (Polsonic, Warsaw, Poland) at 25 °C ± 2 °C (40 kHz frequency). The samples were centrifuged at 13,500 rpm for 15 min for clarification. The resulting supernatants were filtered through 0.2 μm polyethersulfone (PES) syringe filters and stored at 4 °C until LC-MS/MS analysis. Each formula was prepared and analyzed in duplicate. In total, 1 μL of formula extract was used for LC-MS/MS analysis. As the furosine concentration of the samples was significantly higher than that of the other analytes, the extract samples were diluted to fall within the linearity range of the calibration curve.

### 4.5. Chromatographic Separation and MS Conditions

The analytical procedure was adapted from Poojary et al. [30], with some modifications. The analyses were performed using the Vanquish Thermo Scientific liquid chromatograph system. The system consisted of a double split sampler, column thermostat compartment, and double pump and was coupled to a Thermo Scientific OrbiTrap Q Exactive mass spectrometer (Thermo Fisher Scientific Inc., Waltham, MA, USA). Chromatographic separation was achieved on a Phenomenex BioZen 1.7 µm Peptide XB-C18 LC column (150 mm length × 2.1 mm internal diameter × 1.7 μm particle size, Phenomenex, Værløse, Denmark). A multi-step elution gradient was applied, consisting of A: 5 mM PFPA in water and B: 5 mM PFPA in acetonitrile. The mobile phase consisted of the following:

− 0–2 min 100% A;
− 2.01–5 min increase to 20% B;
− 5.01–7 min increase to 25% B (75% A: 25% B);
− 7.01–9 min increase to 30% B (70% A: 30% B);
− 9.01–16 min increase to 35% B (65% A: 35% B);
− 16.01–20 min increase to 40% B (60% A: 35% B);
− 20.01–28 min increase to 100% B;
− 28.01–40 min increase to 100% A.

The flow rate was maintained at 0.250 mL/min. The column compartment and autosampler temperatures were set to 40 °C and 10 °C, respectively. The injection volume was 1 μL of formula extract.

The MS conditions used for AGE determination were as follows:

− ion spray voltage: 3.5 kV;
− ion transfer capillary temperature: 320 °C;
− sheath gas flow rate: 30 arbitrary units;
− auxiliary gas flow rate: 4 arbitrary units;
− sweep gas flow rate: 0 arbitrary units;
− S-lens radio frequency level: 50%.

Full MS scans were acquired over a range of 70–1000 m/z at a resolution of 17,500 full width at half maximum (FWHM). PRM was applied for quantification of the selected glycation products at a resolution of 17,500 FWHM. The automatic gain control (AGC) target was set to 2 × 10^5^, with a maximum injection time of 64 milliseconds. The optimized collision energies for each analyte are listed in Table 1. Data analysis was performed with Thermo Xcalibur software version 4.4.16.14 (Thermo Fisher Scientific Inc., Waltham, MA, USA).

The LC-Orbitrap-MS/MS had a heated electrospray ionization (HESI) source. The system was controlled using Thermo Xcalibur LC Devices 3.2 Robust software (Thermo Fisher Scientific Inc., Waltham, MA, USA).

The mass spectrometer was tuned and calibrated weekly using an electrospray ionization (ESI) positive ion calibration solution. The nitrogen gas for the ion source was produced by the generator Nigen LCMS 40–1 (Claind Brezza, Tremezzina, Italy).

### 4.6. Statistics

The results were summarized using descriptive statistics, including mean, SD, and RSD%. The Mann–Whitney U test was employed to compare the mean content of the tested compounds between the initial and follow-on formulas. For intra-manufacturer comparisons in the initial and follow-on formulas, the Wilcoxon signed-rank test was utilized. All statistical analyses were performed using TIBCO Statistica version 13.3 (TIBCO Software Inc., Palo Alto, CA, USA).

## 5. Conclusions

The applied acid hydrolysis and compound extraction procedure, combined with the LC-Orbitrap-MS/MS method, allowed the determination of concentrations of lysine derivatives and furosine at the level of µg/mL in the ready-to-serve infant formulas.

No statistically significant differences were observed in the concentrations of the analyzed compounds among the formulas from different manufacturers. Similarly, no significant differences were detected when comparing Stage 1 (IF) and Stage 2 (FF) formulas. Importantly, the comparison of liquid and powder products revealed that the liquid formulas contained significantly more CML. This finding emphasizes the importance of proper preparation and storage conditions for ready-to-serve formulas, as well as the influence of external factors, such as outdoor temperature during transportation, on the products’ final composition. The estimated dietary exposure to the tested compounds was approximately 1–2 mg/day, except for furosine, for which the daily exposure reached almost 50 mg/day. Future research will aim to compare these estimates with dietary exposure from breastfeeding by analyzing the content of selected AGEs in human milk samples.

Infant formula constitutes a primary component of daily infant nutrition when breastfeeding is not possible. As shown in our paper, formulas contribute to exposure to dAGEs at an early stage of human life. Therefore, information regarding the potential formation of harmful compounds in these products is crucial, both from a scientific perspective and for safeguarding infant health. Our study provides additional information on concentrations of GOLD and MOLD, the cross-linking AGEs, which are not so commonly studied in infant formulas. Ensuring the safety of infant nutrition during the first months and years of life is therefore of paramount importance. Detailed knowledge of dietary exposure to potentially harmful compounds, based on reliable and highly selective analytical procedures, can lead to further production improvements and minimization of that risk. Therefore, further modifications to the sample preparation procedure are planned to improve the overall analytical process.

## Figures and Tables

**Table 1:** LC-MS/MS parameters of the analyzed compounds.

Compound	Retention Time [min]	Precursor Ion → Product Ion (*m*/*z*)	Collision Energy [eV]
CML	8.52	205.1183 → 84.0806	15
CEL	9.35	219.1339 → 84.0806	15
Furosine	10.66	255.1300 → 84.0806	20
GOLD	11.08	327.2000 → 84.0806	35
MOLD	11.36	341.2000 → 84.0806	37

**Table 2:** Calibration curve ^1^ equations and correlation coefficients (*r*) for the analyzed compounds.

Compound	Calibration Curve Equation	*r*
CML	y = 9098.45x – 482,962	*r* = 0.993
CEL	y = 8278.08x – 484,989	*r* = 0.988
Furosine	y = 8002.84x – 372,779	*r* = 0.990
GOLD	y = 2469.08x – 144,467	*r* = 0.886
MOLD	y = 2270.49x – 144,723	*r* = 0.996

^1^ Recorded in the range of 62.5 ng/mL to 1250 ng/mL.

**Table 3:** Recoveries of the analyzed compounds in infant formula extracts at different spiking levels.

Compound	Recovery (%) for Spiking Level (ng/mL of Infant Formula Extract); n = 6					
	125 ng/mL	RSD [%]	625 ng/mL	RSD [%]	1250 ng/mL	RSD [%]
CML	75.00	1.38	88.43	2.73	80.31	2.30
CEL	88.99	1.20	91.76	3.44	81.23	2.27
Furosine	31.09	2.32	72.47	1.69	71.55	1.67
GOLD	100.00	1.31	121.25	2.54	121.66	2.15
MOLD	79.90	1.27	96.23	2.47	94.45	1.35

**Table 4:** Mean concentrations, standard deviation (SD), and relative standard deviation (RSD%) of the analyzed compounds in the initial and follow-on infant formulas (n = 4 for every sample).

Sample	Values in Milk Samples—Mean ± SD									
	CML [μg/mL]	RSD [%]	CEL [μg/mL]	RSD [%]	Furosine [μg/mL]	RSD [%]	GOLD [μg/mL]	RSD [%]	MOLD [μg/mL]	RSD [%]
Initial Formulas (IF)										
A1 ^L^	3.71 ± 0.55	14.81	1.65 ± 0.04	2.38	39.27 ± 1.36	3.46	1.57 ± 0.06	3.59	1.84 ± 0.02	0.92
B1 ^P^	2.95 ± 0.04	1.29	1.72 ± 0.03	1.92	84.38 ± 2.77	3.28	1.59 ± 0.10	6.23	1.80 ± 0.02	1.18
C1 ^L^	4.99 ± 0.09	1.92	1.74 ± 0.01	0.36	45.39 ± 1.19	2.63	1.67 ± 0.04	2.67	1.76 ± 0.01	0.55
D1 ^L^	3.19 ± 0.14	4.35	1.67 ± 0.01	0.64	57.27 ± 2.12	3.71	1.61 ± 0.07	4.37	1.74 ± 0.04	2.32
E1 ^P^	2.23 ± 0.17	7.65	1.71 ± 0.04	2.09	84.03 ± 5.94	7.07	1.61 ± 0.09	5.92	1.81 ± 0.03	1.41
F1 ^P^	3.90 ± 0.22	5.74	1.71 ± 0.03	1.86	77.58 ± 5.09	6.56	1.57 ± 0.06	3.93	1.66 ± 0.02	1.44
G1 ^P^	2.19 ± 0.02	0.84	1.81 ± 0.11	6.30	87.89 ± 2.01	2.28	1.53 ± 0.01	0.55	1.74 ± 0.03	1.63
H1 ^L^	5.96 ± 0.15	2.49	1.73 ± 0.04	2.22	99.23 ± 10.55	10.64	1.57 ± 0.08	5.07	1.81 ± 0.02	1.26
I1 ^L^	4.04 ± 0.07	1.84	1.70 ± 0.01	0.65	60.32 ± 2.62	4.34	1.66 ± 0.09	5.62	1.82 ± 0.03	1.61
J1 ^P^	2.07 ± 0.19	9.06	1.66 ± 0.02	0.97	55.62 ± 6.29	11.3	1.59 ± 0.05	3.08	1.82 ± 0.02	1.29
Follow-on Formulas (FF)										
A2 ^L^	3.94 ± 0.14	3.66	1.70 ± 0.01	0.60	57.68 ± 0.54	0.94	1.61 ± 0.02	1.44	1.76 ± 0.07	3.73
B2 ^P^	1.82 ± 0.02	1.20	1.73 ± 0.02	1.12	86.51 ± 6.41	7.41	1.52 ± 0.01	0.69	1.82 ± 0.02	1.20
C2 ^P^	1.83 ± 0.05	3.00	1.67 ± 0.12	7.44	54.59 ± 4.47	8.19	1.60 ± 0.12	7.54	1.80 ± 0.04	2.08
D2 ^P^	2.54 ± 0.03	1.19	1.73 ± 0.01	0.60	86.44 ± 1.78	2.06	1.64 ± 0.01	0.67	1.76 ± 0.04	2.41
E2 ^P^	2.35 ± 0.05	1.95	1.72 ± 0.06	3.22	102.35 ± 7.40	7.23	1.61 ± 0.10	6.34	1.84 ± 0.05	2.52
F2 ^P^	4.06 ± 0.17	4.25	1.73 ± 0.02	0.92	86.87 ± 4.98	5.73	1.56 ± 0.04	2.48	1.71 ± 0.03	1.66
G2 ^P^	1.95 ± 0.03	1.50	1.61 ± 0.01	0.62	61.63 ± 2.62	4.25	1.51 ± 0.02	1.17	1.75 ± 0.03	1.55
H2 ^L^	6.27 ± 0.47	7.47	1.71 ± 0.02	0.89	77.12 ± 3.36	4.36	1.55 ± 0.09	5.56	1.80 ± 0.08	4.37
I2 ^P^	2.45 ± 0.34	13.87	1.66 ± 0.02	0.98	67.42 ± 4.14	6.14	1.56 ± 0.06	3.74	1.84 ± 0.01	0.37
J2 ^P^	4.61 ± 0.12	2.39	1.85 ± 0.03	1.37	96.56 ± 1.94	2.01	1.56 ± 0.02	1.58	1.72 ± 0.03	1.77

^L^ The milk was a ready-to-serve liquid; ^P^ the milk was a powder requiring dissolving in water.

**Table 5:** Descriptive statistics (mean, range, and protein-normalized concentrations) for the analyzed glycation products in ready-to-serve infant formulas.

Compound	Formula	Mean [μg/mL of Milk]	Min–Max Range [μg/mL of Milk]	Mean Concentration [mg/100 g of Protein in Milk]
CML	IF	3.52	2.07–5.96	25.83
	FF	3.18	1.82–6.27	22.59
CEL	IF	1.61	0.81–1.74	11.81
	FF	1.71	1.61–1.85	12.14
Furosine	IF	69.10	39.27–99.23	506.97
	FF	77.72	54.59–102.35	551.99
GOLD	IF	1.60	1.53–1.67	11.74
	FF	1.57	1.51–1.64	11.15
MOLD	IF	1.78	1.66–1.84	13.06
	FF	1.78	1.71–1.84	12.64

**Table 6:** Energy value and nutrient content in the tested infant formulas, based on manufacturer-provided data from product labels.

Sample	Value per 100 mL of Ready-to-Serve Formula			
	Energy Value [kcal]	Protein [g]	Total Carbohydrates (Mono- and Disaccharides) [g]	Lipids [g]
Initial Formulas (IF)				
A1 ^L^	66	1.3	7.2 (7.0)	3.4
B1 ^P^	67	1.3	8.2 (7.7)	3.3
C1 ^L^	66	1.3	7.5 (7.4)	3.4
D1 ^L^	65	1.2	7.6 (7.6)	3.2
E1 ^P^	66	1.3	7.3 (7.2)	3.4
F1 ^P^	66	1.5	7.2 (7.0)	3.4
G1 ^P^	68	1.88	7.4 (0.77)	3.5
H1 ^L^	67	1.24	7.5 (7.3)	3.5
I1 ^L^	66	1.3	7.0 (7.0)	3.6
J1 ^P^	66	1.3	7.3 (7.2)	3.4
Follow-on Formulas (FF)				
A2 ^L^	68	1.4	8.2 (8.1)	3.2
B2 ^P^	67	1.3	8.2 (7.4)	3.2
C2 ^P^	68	1.4	8.3 (8.2)	3.2
D2 ^P^	66	1.3	7.8 (7.6)	3.3
E2 ^P^	68	1.4	8.1 (8.0)	3.2
F2 ^P^	68	1.5	7.7 (7.5)	3.3
G2 ^P^	68	1.68	7.7 (3.5)	3.4
H2 ^L^	67	1.1	8.3 (8.3)	3.2
I2 ^P^	68	1.4	8.1 (8.0)	3.2
J2 ^P^	68	1.6	8.1 (3.5)	3.1

^L^ The milk was a ready-to-serve liquid; ^P^ the milk was a powder requiring dissolving in water.

## Data Availability

Dataset available on request from the authors.

## Supplementary Materials

The following supporting information can be downloaded at: https://www.mdpi.com/article/10.3390/molecules30132753/s1: Table S1: Detailed information on the infant formula content, based on data from product labels. Table S2: Mean ± SD concentrations of the tested compounds in the formula extracts injected into the chromatograph (n = 4 for each sample). Figure S1: Calibration graphs used for the quantification of furosine and AGEs in infant formula extracts. Figure S2: Representative LC-Orbitrap-MS/MS chromatogram (left) and mass spectrum (right) for the determination of CML in infant formula extract sample H2 (recorded in PRM mode). Figure S3: LC-Orbitrap-MS/MS chromatogram (left) and mass spectrum (right) recorded in PRM mode for CEL determined in an infant formula extract sample H2. Figure S4: LC-Orbitrap-MS/MS chromatogram (left) and mass spectrum (right) recorded in PRM mode for furosine determined in an infant formula extract sample H2. Figure S5: LC-Orbitrap-MS/MS chromatogram (left) and mass spectrum (right) recorded in PRM mode for GOLD determined in an infant formula extract sample H2. Figure S6: LC-Orbitrap-MS/MS chromatogram (left) and mass spectrum (right) recorded in PRM mode for MOLD determined in an infant formula extract sample H2.

## Abbreviations

The following abbreviations are used in this manuscript:

1-DG	1-deoxyglucosone
3-DG	3-deoxyglucosone
AGC	automatic gain control
AGEs	advanced glycation end-products
CEA	N^ε^-carboxyethylarginine
CEL	N^ε^-carboxyethyllysine
CMA	N^ε^-carboxymethylarginine
CML	N^ε^-carboxymethyllysine
dAGEs	dietary advanced glycation end-products
EFSA	European Food Safety Authority
EGPs	early glycation products
ELISA	enzyme-linked immunosorbent assay
ESI	electrospray ionization
FF	follow-on formula
FWHM	full width at half maximum
GOLD	glyoxal lysine dimer
HESI	heated electrospray ionization
IF	initial formula
IGPs	intermediate glycation products
LC	liquid chromatography
LOD	limit of detection
LOQ	limit of quantification
MG-H1	methylglyoxal-derived hydroimidazolone
MGO	methylglyoxal
MOLD	methylglyoxal lysine dimer
MRPs	Maillard reaction products
MS	mass spectrometry
MS/MS	tandem mass spectrometry
PES	polyethersulfone
PFPA	perfluoropropionic anhydride
PRM	parallel reaction monitoring
RAGE	receptor for advanced glycation end-products
RSD%	relative standard deviation
SD	standard deviation
S/N	signal-to-noise
TAGEs	toxic advanced glycation end-products
UHT	ultra-high temperature
