# Supplementary material for: LC-Orbitrap-MS/MS Analysis of Chosen Glycation Products in Infant Formulas
Source: Molecules. 2025 Jun 26;30(13):2753. doi: 10.3390/molecules30132753 (PMC12250656; doi:10.3390/molecules30132753)
Supplement: Supplementary file 1 [file molecules-30-02753-s001.zip › Figure S1.pdf]

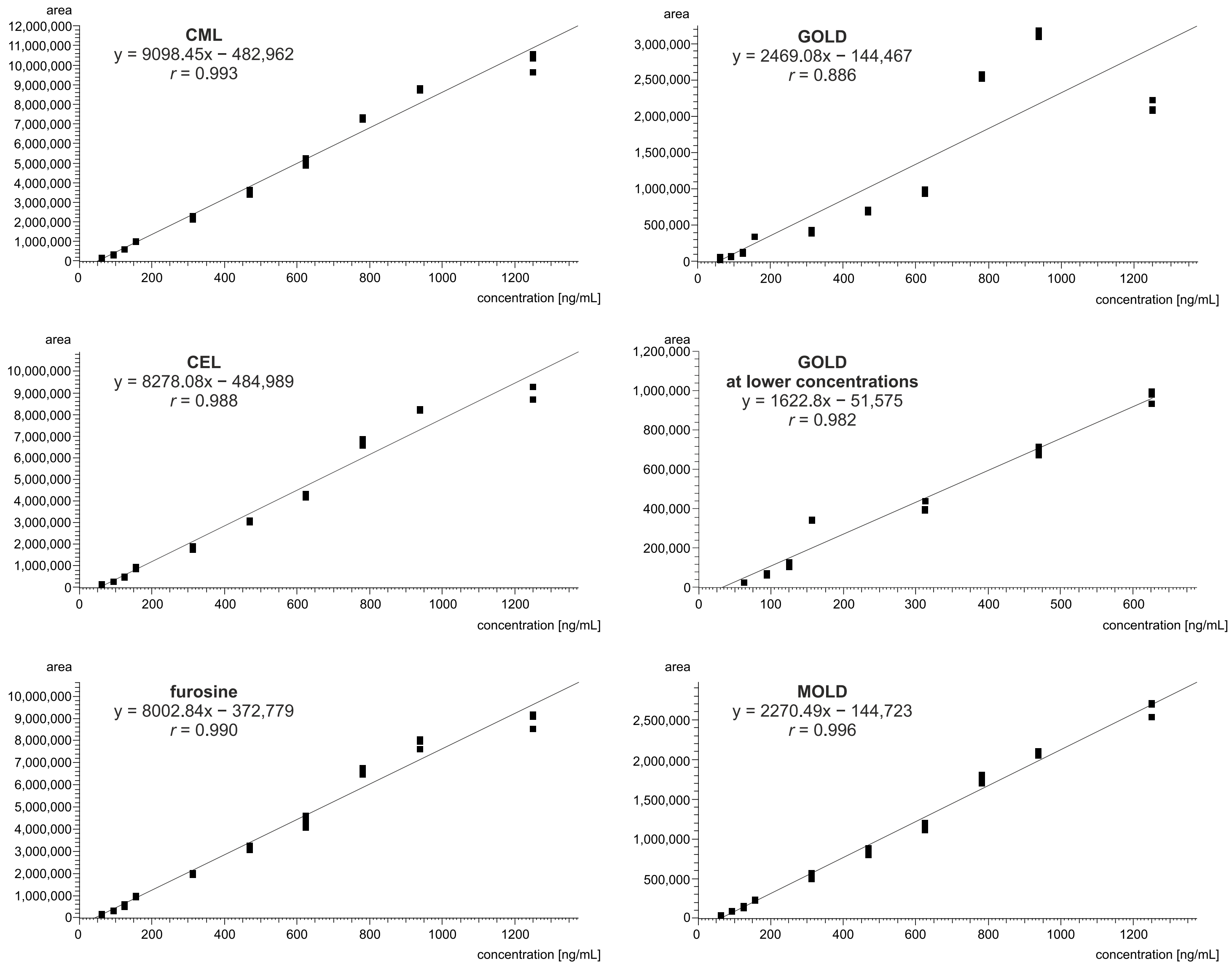

Figure S1. Calibration graphs used for quantification of furosine and AGEs in infant formula extracts.
