# Supplementary material for: LC-Orbitrap-MS/MS Analysis of Chosen Glycation Products in Infant Formulas
Source: Molecules. 2025 Jun 26;30(13):2753. doi: 10.3390/molecules30132753 (PMC12250656; doi:10.3390/molecules30132753)
Supplement: Supplementary file 1 [file molecules-30-02753-s001.zip › Figures S2-S6.pdf]

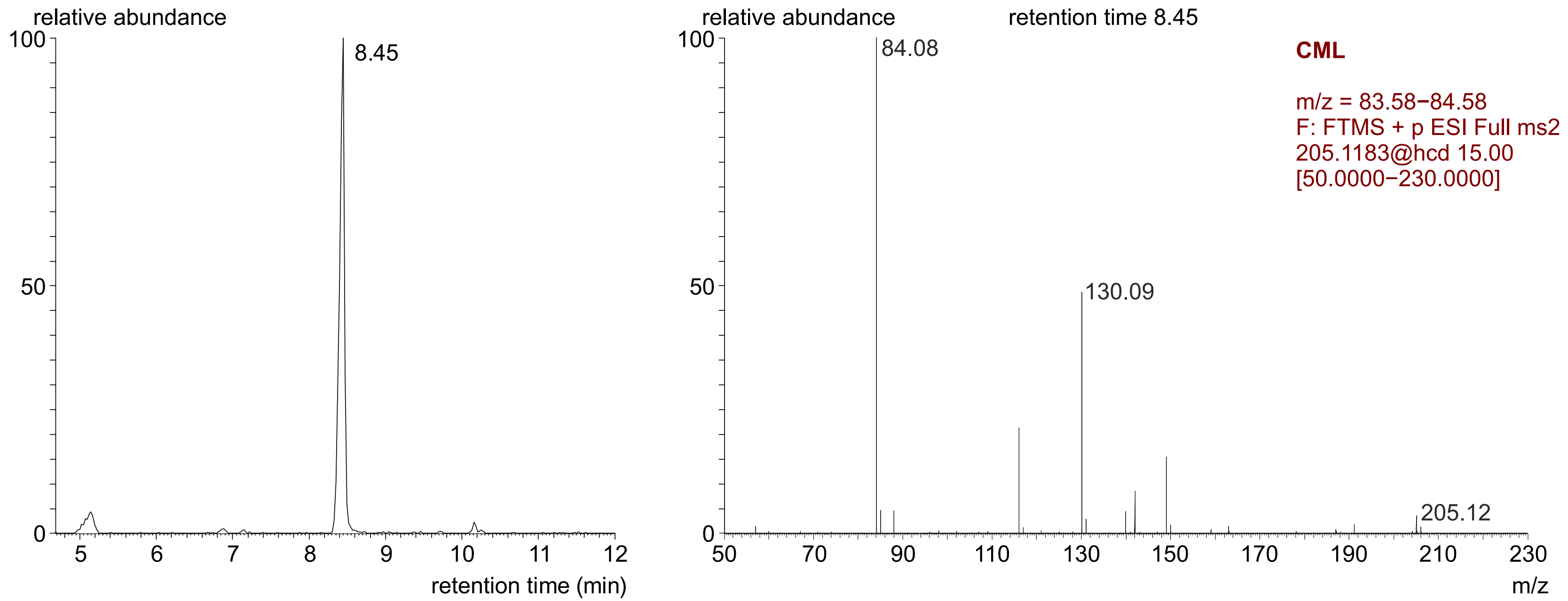

Figure S2. Representative LC-Orbitrap-MS/MS chromatogram (left) and mass spectrum (right) for determination of CML in infant formula extract sample H2 (recorded in PRM mode).

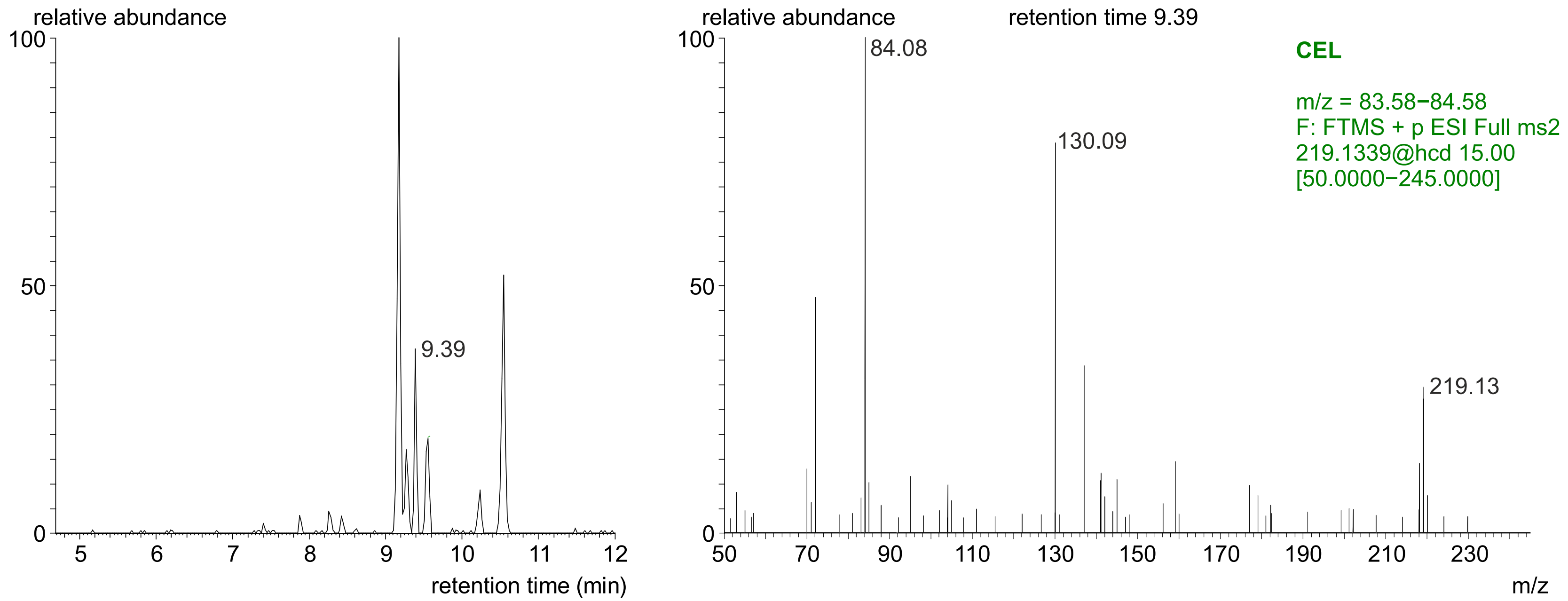

Figure S3. Representative LC-Orbitrap-MS/MS chromatogram (left) and mass spectrum (right) for determination of CEL in infant formula extract sample H2 (recorded in PRM mode).

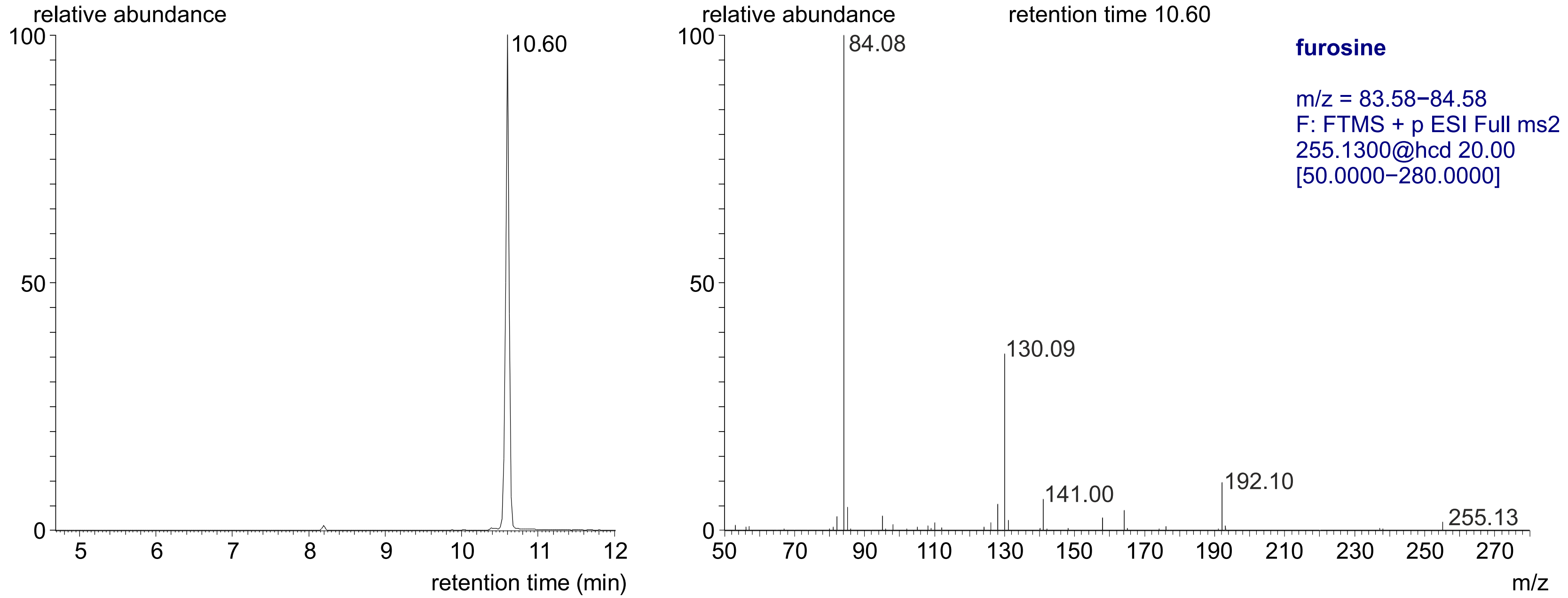

Figure S4. Representative LC-Orbitrap-MS/MS chromatogram (left) and mass spectrum (right) for determination of furosine in infant formula extract sample H2 (recorded in PRM mode).

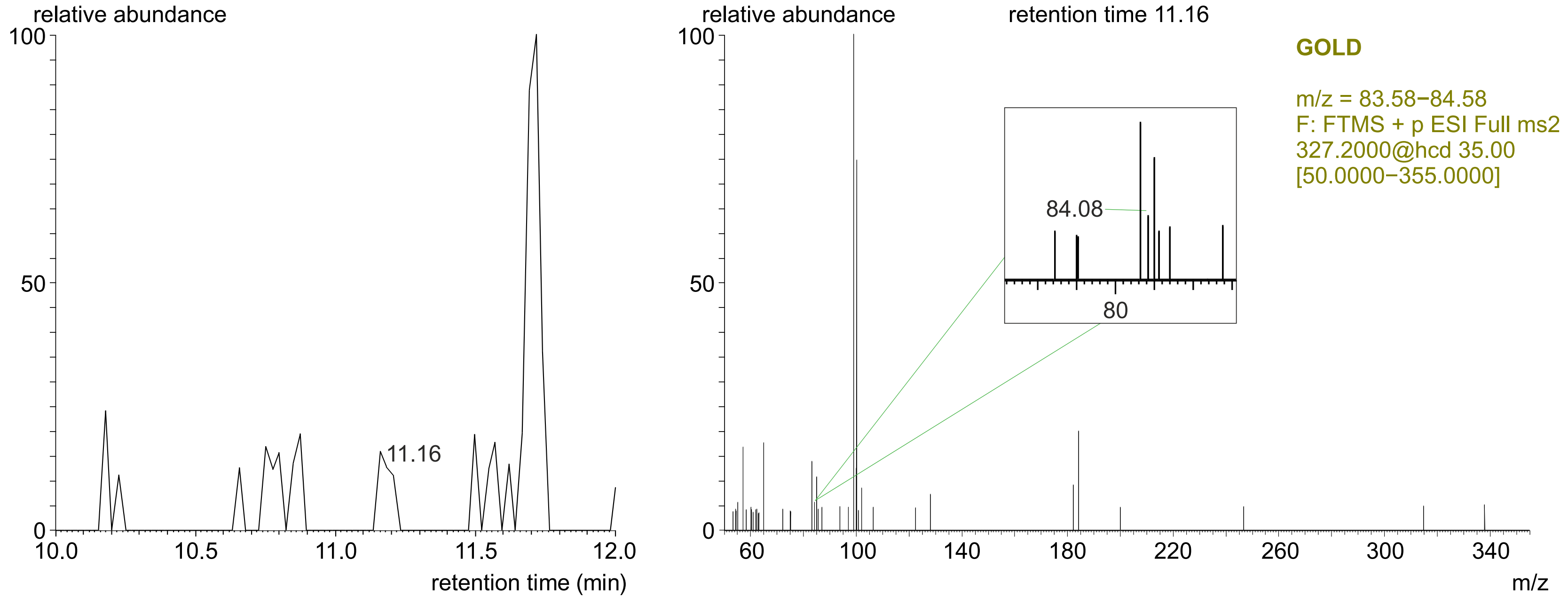

Figure S5. Representative LC-Orbitrap-MS/MS chromatogram (left) and mass spectrum (right) for determination of GOLD in infant formula extract sample H2 (recorded in PRM mode).

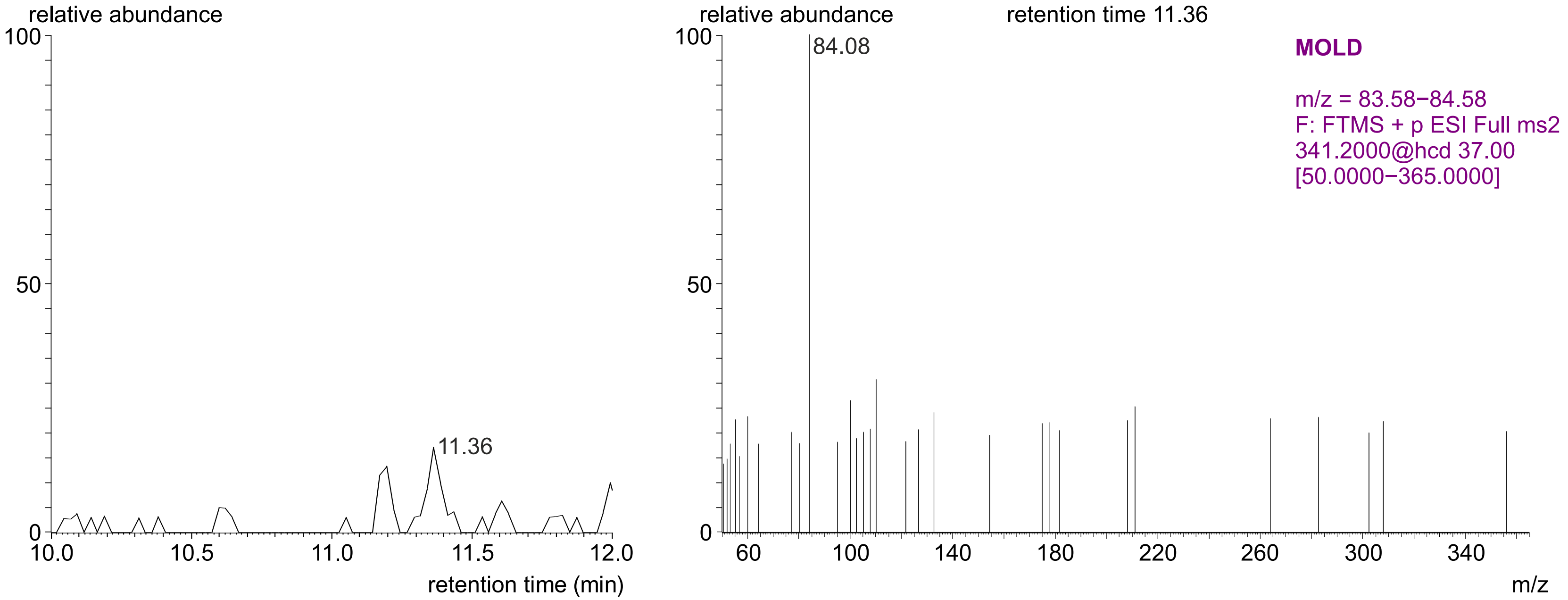

Figure S6. Representative LC-Orbitrap-MS/MS chromatogram (left) and mass spectrum (right) for determination of MOLD in infant formula extract sample H2 (recorded in PRM mode).
