# Supplementary material for: LC-Orbitrap-MS/MS Analysis of Chosen Glycation Products in Infant Formulas
Source: Molecules. 2025 Jun 26;30(13):2753. doi: 10.3390/molecules30132753 (PMC12250656; doi:10.3390/molecules30132753)
Supplement: Supplementary file 1 [file molecules-30-02753-s001.zip › Table S1.pdf]

Table S1. Detailed information on the infant formula content, based on data from product labels.

Sample	Value per 100 mL of Ready-to-Serve Product									
	Energy Value [kcal]	Protein [g]	Total Carbohydrate [g]	Total Sugars [g]	Dietary Fiber [g]	GOS [g]	FOS [g]	Total Lipids [g]	Saturated Fatty Acids [g]	Sodium [mg]
**Initial Formulas (IF)**										
**A1** [L]	66	1.3	7.2	7.0	0.6	0.52	0.08	3.4	1.6	24.0
**B1** [P]	67	1.3	8.2	7.7	0.0	-	-	3.3	1.2	27.9
**C1** [L]	66	1.3	7.5	7.4	0.3	0.24	0.04	3.4	1.0	23.0
**D1** [L]	65	1.2	7.6	7.6	0.3	0.3	-	3.2	1.3	27.0
**E1** [P]	66	1.3	7.3	7.2	0.7	0.48	0.08	3.4	1.6	21.9
**F1** [P]	66	1.5	7.2	7.0	0.6	0.48	0.08	3.4	1.2	33.0
**G1** [P]	68	1.88	7.4	0.77	-	-	-	3.5	0.54	32
**H1** [L]	67	1.24	7.5	7.3	0.15	-	-	3.5	0.3	21.3
**I1** [L]	66	1.3	7.0	7.0	0.3	0.3	-	3.6	1.6	20
**J1** [P]	66	1.3	7.3	7.2	0.7	0.24	0.04	3.4	1.5	22
**Follow-on Formulas (FF)**										
**A2** [L]	68	1.4	8.2	8.1	0.6	0.48	0.08	3.2	1.0	28
**B2** [P]	67	1.3	8.2	7.4	0.0	-	-	3.2	1.2	27.7
**C2** [P]	68	1.4	8.3	8.2	0.3	0.24	0.04	3.2	1.0	23.0
**D2** [P]	66	1.3	7.8	7.6	0.46	0.42	-	3.3	1.2	27.9
**E2** [P]	68	1.4	8.1	8.0	0.6	0.48	0.08	3.2	1.5	23.0
**F2** [P]	68	1.5	7.7	7.5	0.6	0.48	0.08	3.3	1.2	33.9
**G2** [P]	68	1.68	7.7	3.5	-	-	-	3.4	1.41	24
**H2** [L]	67	1.1	8.3	8.3	0.05	-	-	3.2	0.3	32.3
**I2** [P]	68	1.4	8.1	8.0	0.6	0.48	0.08	3.2	1.5	23.0
**J2** [P]	68	1.6	8.1	3.5	0.6	0.49	0.08	3.1	1.3	30.0

[L] milk was a ready-to-serve liquid; [P] milk was a powder requiring dissolving in water

GOS—galactooligosaccharide

FOS—fructooligosaccharide
