# Supplementary material for: LC-Orbitrap-MS/MS Analysis of Chosen Glycation Products in Infant Formulas
Source: Molecules. 2025 Jun 26;30(13):2753. doi: 10.3390/molecules30132753 (PMC12250656; doi:10.3390/molecules30132753)
Supplement: Supplementary file 1 [file molecules-30-02753-s001.zip › Table S2.pdf]

Table S2. Mean ± SD concentrations of the tested compounds in the formula extracts injected into the chromatograph (n = 4 for each sample).

Sample	CML [ng/mL]	CEL [ng/mL]	Furosine [ng/mL]	GOLD [ng/mL]	MOLD [ng/mL]
			**Initial Formulas (IF)**		
**A1** [L]	148.3 ± 19.0	66.0 ± 1.4	1570.7 ± 47.1	62.6 ± 1.9	73.4 ± 0.6
**B1** [P]	117.9 ± 1.3	68.9 ± 1.1	3375.1 ± 95.3	63.8 ± 3.4	72.1 ± 0.7
**C1** [L]	199.8 ± 3.3	69.5 ± 0.2	1815.8 ± 41.4	66.7 ± 1.5	70.3 ± 0.3
**D1** [L]	127.5 ± 4.8	66.8 ± 0.4	2290.9 ± 73.6	64.2 ± 2.4	69.8 ± 1.4
**E1** [P]	89.0 ± 5.9	68.6 ± 1.2	3361.1 ± 205.7	64.2 ± 3.3	72.3 ± 0.9
**F1** [P]	156.0 ± 7.7	68.5 ± 1.1	3103.0 ± 176.3	62.8 ± 2.1	66.4 ± 0.8
**G1** [P]	87.74 ± 0.74	72.3 ± 4.0	3515.7 ± 69.6	61.2 ± 0.3	69.7 ± 1.0
**H1** [L]	238.6 ± 5.1	69.2 ± 1.3	3969.3 ± 365.6	62.9 ± 2.8	72.4 ± 0.8
**I1** [L]	161.5 ± 2.6	68.0 ± 0.4	2412.9 ± 90.6	66.5 ± 3.2	72.7 ± 1.0
**J1** [P]	82.8 ± 6.5	66.3 ± 0.6	2224.6 ± 217.8	63.7 ± 1.7	72.9 ± 0.8
			**Follow-on Formulas (FF)**		
**A2** [L]	157.7 ± 5.0	68.0 ± 0.4	2307.2 ± 18.9	64.4 ± 0.8	70.6 ± 2.3
**B2** [P]	119.5 ± 1.1	69.3 ± 0.7	3460.5 ± 222.0	60.8 ± 0.4	72.7 ± 0.8
**C2** [P]	73.1 ± 1.9	66.7 ± 4.3	2183.5 ± 154.8	64.0 ± 4.2	72.1 ± 1.3
**D2** [P]	101.7 ± 1.0	69.1 ± 0.4	3457.5 ± 61.7	65.4 ± 0.4	70.4 ± 1.5
**E2** [P]	94.2 ± 1.6	68.7 ± 2.0	4094.1 ± 256.4	64.4 ± 3.5	73.7 ± 1.6
**F2** [P]	162.2 ± 6.0	69.1 ± 0.5	3474.7 ± 172.5	62.3 ± 1.3	68.6 ± 1.0
**G2** [P]	78.0 ± 1.0	64.7 ± 0.3	2465.3 ± 90.7	60.3 ± 0.6	69.8 ± 0.9
**H2** [L]	250.7 ± 16.2	68.4 ± 0.5	3084.7 ± 116.5	62.2 ± 3.0	72.0 ± 2.7
**I2** [P]	97.9 ± 11.8	66.3 ± 0.6	2696.9 ± 143.4	62.5 ± 2.0	73.8 ± 0.2
**J2** [P]	184.3 ± 3.8	74.0 ± 0.9	3862.3 ± 67.1	62.3 ± 0.9	68.9 ± 1.1

[L] milk was a ready to serve liquid; [P] milk was a powder requiring dissolving in water
